# GPA33 expression in colorectal cancer can be induced by WNT inhibition and targeted by cellular therapy

**DOI:** 10.1038/s41388-024-03200-3

**Published:** 2024-10-29

**Authors:** Teresa Börding, Tobias Janik, Philip Bischoff, Markus Morkel, Christine Sers, David Horst

**Affiliations:** 1 Institute of Pathology, Charité – Universitätsmedizin Berlin, Corporate Member of Freie Universität Berlin and Humboldt-Universität zu Berlin, Berlin, Germany; 2 German Cancer Consortium (DKTK) Partner Site Berlin, German Cancer Research Center (DKFZ), Heidelberg, Germany; 3 Berlin Institute of Health at Charité – Universitätsmedizin Berlin, BIH Biomedical Innovation Academy, BIH Charité Clinician Scientist Program, Berlin, Germany

**Keywords:** Colorectal cancer, Immunotherapy, Cancer immunotherapy, Targeted therapies, Tumour heterogeneity

## Abstract

GPA33 is a promising surface antigen for targeted therapy in colorectal cancer (CRC). It is expressed almost exclusively in CRC and intestinal epithelia. However, previous clinical studies have not achieved expected response rates. We investigated GPA33 expression and regulation in CRC and developed a GPA33-targeted cellular therapy. We examined GPA33 expression in CRC cohorts using immunohistochemistry and immunofluorescence. We analyzed GPA33 regulation by interference with oncogenic signaling in vitro and in vivo using inhibitors and conditional inducible regulators. Furthermore, we engineered anti-GPA33-CAR T cells and assessed their activity in vitro and in vivo. GPA33 expression showed consistent intratumoral heterogeneity in CRC with antigen loss at the infiltrative tumor edge. This pattern was preserved at metastatic sites. GPA33-positive cells had a differentiated phenotype and low WNT activity. Low GPA33 expression levels were linked to tumor progression in patients with CRC. Downregulation of WNT activity induced GPA33 expression in vitro and in GPA33-negative tumor cell subpopulations in xenografts. GPA33-CAR T cells were activated in response to GPA33 and reduced xenograft growth in mice after intratumoral application. GPA33-targeted therapy may be improved by simultaneous WNT inhibition to enhance GPA33 expression. Furthermore, GPA33 is a promising target for cellular immunotherapy in CRC.

## Introduction

Colorectal cancer (CRC) ranks second among gender-nonspecific cancers in terms of both incidence and mortality [1]. Most colorectal adenocarcinomas originate from the acquisition of driver mutations in *APC*, followed by sequential accumulation of further mutations in key genes such as *KRAS*, *PIK3CA*, and *TP53*, which are critical for tumor progression and malignancy [2]. Collectively, these mutations lead to the activation of a signaling network of oncogenic pathways, including WNT- and MAPK-signaling. Despite their intrinsic activation, these oncogenic signals may display intratumoral heterogeneity. For instance, varying levels of WNT activity are observed within a single CRC despite all tumor cells carrying an identical driver mutation in APC [3]. Here, higher WNT activity is observed at the infiltrative tumor edge, which is often associated with cells undergoing epithelial-mesenchymal transition (EMT). These cells are linked to an undifferentiated stem cell-like phenotype and cancer progression [4, 5]. This indicates that cancer cell heterogeneity may be a roadblock to therapeutic success due to phenotypic plasticity and heterogeneous susceptibility to therapy.

Treatment of advanced CRC often includes chemotherapy to fight systemically spread cancer cells in tumor metastases. As an alternative to chemotherapy, therapeutic approaches have been tailored to recognize cancer cell-specific antigens which may be targeted by therapeutic antibodies. However, potent anti-cancer activity may require additional functionality of tumor directed antibodies, such as enhanced engagement of the cellular immune system, drug conjugates, or radiolabels [6]. Recently, chimeric antigen receptor (CAR) T cells have emerged as a potent and highly sensitive adoptive immunotherapeutic approach with great success in hematological malignancies [7]. While conventional T cell receptors require major histocompatibility complex (MHC)-dependent activation, patient-derived CAR T cells can be activated by different types of targets expressed on the tumor cell surface [8]. However, suitable antigens for adoptive cell therapy of solid tumors are rare, as they need to be highly specific for cancer cells and accessible to CAR T cells [9].

Glycoprotein A33 (GPA33) is a glycosylated plasma membrane protein of the immunoglobulin family with unknown function and is expressed almost exclusively in epithelial cells of intestinal tissues and in 95% of CRCs [10, 11]. Due to its tissue-specific expression pattern on the plasma membrane surface, GPA33 has become a promising antigen for targeted therapy of CRC. Some in vivo studies and clinical trials have used humanized antibodies and antibody fragments targeting GPA33 [12–15]. While in some clinical trials stable disease was achieved in individual patients, predefined objective response rates were not sufficiently met. This suggests that there may be an unidentified resistance mechanism against GPA33 targeted therapy, possibly due to treatment resistant tumor cell subpopulations.

In this study we analyzed the expression pattern and regulation of GPA33 in CRC. We identify WNT-active, poorly differentiated cancer cells as a potential tumor cell subpopulation evading GPA33-targeted therapy. We further show that GPA33 can be induced in antigen-negative CRC cell subpopulations using WNT inhibitors in vitro and in vivo. Finally, we introduce a GPA33-directed CAR T cell therapy with promising antitumor efficacy.

## Results

### GPA33 expression is lost in undifferentiated WNT-active cells at the infiltrative tumor edge

**Fig. 1:**
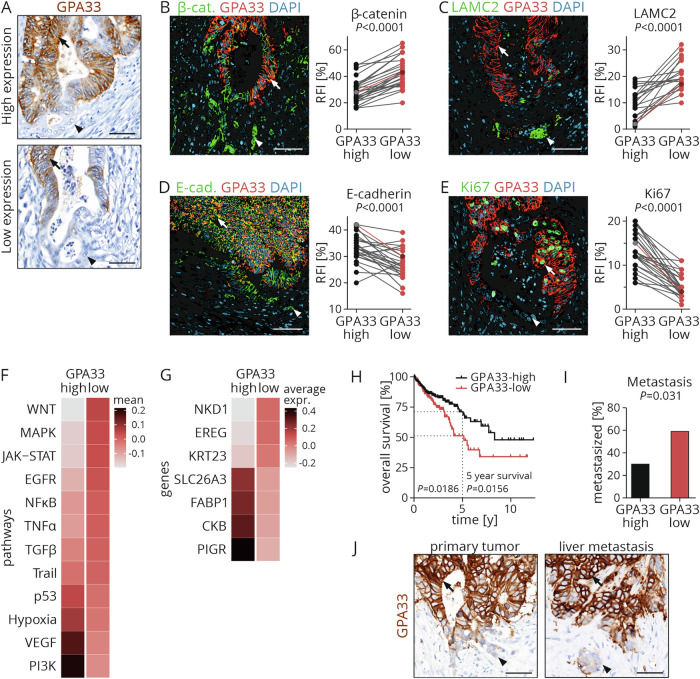
GPA33 expression is lost in undifferentiated WNT-active tumor cells and is associated with favorable prognosis. **A** Representative immunostaining for GPA33 in primary colon cancer tissues with high and low GPA33 expression levels in the collection of UICC stage II patients. Arrows indicate GPA33-positive and arrowheads GPA33-negative tumor cells. (B-E) Representative double immunofluorescence staining and quantification of co-immunofluorescence for β-catenin (**B**), LAMC2 (**C**), E-cadherin (**D**) and Ki67 (**E**) in primary colon cancer tissue. Arrows indicate GPA33-positive and arrowheads GPA33-negative tumor cells. Mean relative fluorescence intensity (RFI) for the indicated proteins in tumor cells with high (upper quartile) and low (lower quartile) GPA33 staining intensity are shown. Data were derived from 13 CRC cases (LAMC2, n = 8; the other five tumors showed no LAMC2-positive cells). For each case, at least two TMA cores from different tumor regions were quantified using at least n ≥ 100 tumor cells; two-tailed paired t-test. Cases with highlighted data points are shown in images. **F** Analysis of signaling pathway activity in the scRNA-seq data using the Progeny package. The integrated dataset was subset into *GPA33*-high and *GPA33*-low groups based on average *GPA33* expression >1. **G** Differentially expressed genes between the *GPA33*-high and *GPA33*-low subsets. Analyses were performed using the FindAllMarkers function of the Seurat package. **H** Kaplan-Meier curve for overall survival of patients with *GPA33*-high and *GPA33*-low RNA expression levels in the TCGA-COAD cohort; log-rank test for overall survival and chi-square test for 5-year survival. **I** Association between GPA33 protein expression levels and liver metastasis in a matched case-control collection of 64 colon cancers; chi-square test. **J** Representative immunostaining of GPA33 in the primary tumor and liver metastasis of the same CRC patient. Arrows indicate GPA33-positive and arrowheads GPA33-negative tumor cells. Scale bars: 50 µm.

To assess GPA33 expression in CRC, we first investigated a collection of 223 primary UICC stage II CRC cases by immunhistochemistry (Table S1). While 98.2% of these cases showed positive membranous staining of tumor cells (Fig. 1A), we found a substantial heterogeneity of GPA33 expression at the intratumoral cell-to-cell level (Fig. S1A). A subpopulation of GPA33-negative tumor cells was found in 95.0% of CRCs. Importantly, GPA33-loss in tumor cells generally occurred in a stereotypic pattern towards the infiltrative tumor edge (Fig. 1A). Stromal cells were generally GPA33-negative.

To further characterize tumor cells with and without GPA33 expression, we analyzed a subset of 14 CRC cases using double immunofluorescence and confocal microscopy for key cell state markers of WNT activity (β-catenin), EMT (LAMC2), differentiation (E-cadherin), and proliferation (Ki67) (Fig. 1B*–*E). We quantified co-immunofluorescence of these antigens with GPA33 and found that GPA33-negative cancer cells displayed high levels of nuclear β-catenin, indicating high WNT activity (Fig. 1B), and strong expression of the EMT marker LAMC2 (Fig. 1C). In contrast, GPA33-positive cancer cells were generally positive for E-cadherin, a marker for tumor cell differentiation (Fig. 1D). Notably, proliferation was higher in the GPA33-positive cancer cell population than in GPA33-negative cancer cells, as indicated by Ki67 (Fig. 1E).

Next, we assessed differences in signaling pathway activities of colon cancer cell subpopulations with differential *GPA33* mRNA expression, using single-cell transcriptome sequencing data of 12 samples of freshly resected colon cancers [16]. We grouped cancer cells into *GPA33*-high and -low clusters and looked for differences in pathway activities through assessment of expression of specific target gene signatures (Fig. 1F). We observed the strongest mean difference in signaling pathway activity for WNT, which was downregulated in the *GPA33*-high tumor cell population. *GPA33*-high cells were also less active in MAPK and JAK-STAT signaling but conversely showed strong activation of PI3K signaling. Looking at the most differentially expressed genes between the two groups, we found high expression of *NKD1*, *EREG* and *KRT23* in *GPA33*-low cells (Fig. 1G), suggesting a link to WNT activity [17], MAPK signaling [18] and stemness [19], respectively. Conversely, gene expressions previously linked to tumor cell differentiation and favorable outcome, such as *SLC26A3*, *PIGR*, *CKB* and *FABP1* [20–23], were highly expressed in GPA33-high cancer cells.

These results demonstrate that the expression of GPA33 shows strong intratumoral heterogeneity in CRC and is lost in undifferentiated subsets of tumor cells with high oncogenic signaling activity that may be primed to undergo EMT at the infiltrative tumor edge.

### High GPA33 expression is associated with favorable outcome in CRC

We then looked for associations of GPA33 protein expression and clinical outcome in CRC. In UICC stage II CRC, we observed no significant association with tumor specific survival (data not shown). However, when we analyzed the COAD cohort profiled by The Cancer Genome Atlas (TCGA), consisting of 438 CRC patients of all stages with annotated survival status, Kaplan-Meier analysis revealed that low *GPA33* mRNA expression was significantly associated with poor overall survival (Fig. 1H). This association was independent of microsatellite status (Fig. S1B, C). Furthermore, we analyzed GPA33 protein expression in a case-control collection of 32 matched pairs of metastatic and non-metastatic colorectal cancers (Table S2). In this collection, we found a significant correlation between low GPA33 expression and liver metastasis (Fig. 1I). While only 30.0% of the tumors with high GPA33 expression had metastasized, 59.1% of the cases with low GPA33 expression had developed distant liver metastases. This correlation was also significant when analyzing cases with proficient mismatch repair status (pMMR) only (Fig. S1D). Importantly, when we analyzed six individual pairs of primary colon cancers and corresponding liver metastases, expression and distribution patterns of GPA33-positive and -negative tumor cells with loss of GPA33 at the infiltrative tumor edge were preserved between primary tumors and metastases (Fig. 1J). These data suggest that GPA33 expression is inversely linked to colon cancer progression and metastasis in advanced stage disease. Furthermore, the heterogeneous intratumoral distribution of GPA33 protein is a stable phenotype that also occurs at metastatic sites.

### GPA33 expression depends on cell density and WNT signaling activity

**Fig. 2:**
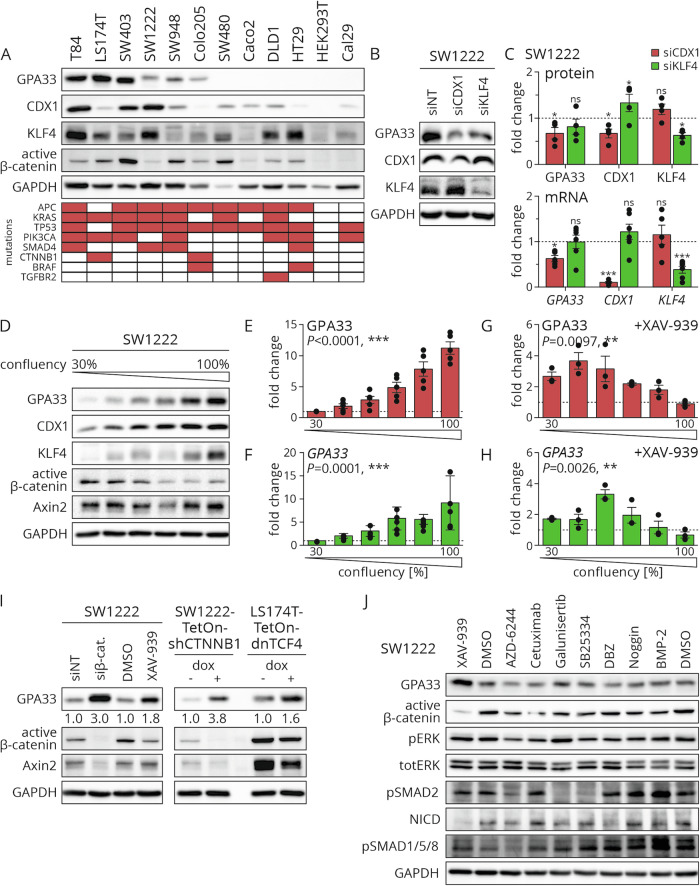
GPA33 expression can be induced through WNT inhibition in vitro. **A** Immunoblot for the indicated antigens in various cell lines treated with DMSO for 72 h and harvested at 50% confluency. The bottom panel indicates known driver mutations in these cell lines; red = mutated, white = wildtype. **B**, **C** SW1222 cells were treated with control (siNT), siCDX1, or siKLF4 for 72 h. **B** Representative immunoblots of the indicated antigens. **C** Quantification of fold change of protein (n = 4) and mRNA (n = 6) levels upon treatment with indicated siRNA, normalized to control, mean ± SEM; 2-way ANOVA. **D**–**F** SW1222 cells were treated with DMSO for 72 h and confluency at harvest was documented by qualitative assessment using light microscopy. **D** Representative immunoblot of protein lysates for the indicated proteins and cell confluence. Quantification of fold change of GPA33 protein (**E**) and mRNA (**F**) normalized to lowest cell confluency, n = 6, mean ± SEM; 1-way ANOVA. **G**, **H** SW1222 cells were treated with 10 µM XAV-939 or control (DMSO) for 72 h at varying cell confluencies. Quantification of fold-change of GPA33 protein (**G**) and mRNA (**H**) normalized to control, n = 3, mean ± SEM; 1-way ANOVA. **I** Immunoblot for the indicated antigens. The indicated cell lines were treated with control (siNT, DMSO, H_2_O), siβ‑catenin, XAV-939, or doxycycline for 72 h and harvested at 50% cell confluency. Values below the GPA33 blot represent fold-induction to control, normalized to GAPDH. **J** Immunoblot for indicated antigens. SW1222 cells were treated with control (DMSO) or the indicated drugs for 72 h and harvested at 50% confluency. *, *P* < 0.05; **P < 0.01; ***P < 0.001.

In order to investigate the biologic basis of differential GPA33 expression, we analyzed GPA33 protein levels in a panel of 12 cell lines. While two non-CRC cell lines HEK293T and Cal29 were negative for GPA33, six out of ten CRC cell lines expressed this antigen at varying levels (Fig. 2A). Expression did not appear to depend on the presence of a specific typical CRC driver mutation in these cell lines (Fig. 2A). We then assessed the expression of the transcription factors CDX1 and KLF4, both of which were previously suggested regulators of GPA33 [24, 25]. High levels of CDX1 were only expressed in GPA33-positive cell lines, while there was no clear association of GPA33 expression and KLF4 (Fig. 2A). Next, we depleted CDX1 and KLF4 in SW1222 colon cancer cells by siRNA. Knockdown of CDX1 but not of KLF4 resulted in a significant reduction in GPA33 protein and RNA levels (Fig. 2B, C). Simultaneous knockdown of both transcription factors showed similar effects to depletion of CDX1 alone (Fig. S2A).

Next, we investigated whether different culture conditions affected GPA33 expression. In SW1222 cells, GPA33 protein and mRNA expression strongly depended on cellular confluency and ranged from low to high expression levels in low and high culture density, respectively (Fig. 2D–F). Under changing growth densities, GPA33 expression mirrored that of CDX1 and KLF4, and showed opposite changes with presence of active β-catenin (Fig. 2D) and expression of the WNT/β-catenin target gene and stem cell marker *LGR5* (Fig. S2B). Furthermore, testing the impact of tumor cell density on two other CRC cell lines T84 and SW403 showed similar effects on GPA33 levels (Fig. S2C). These results indicate that GPA33 expression is induced by tumor cell differentiation under increasing culture density. Its expression can therefore be modulated in vitro. Interestingly, high density culture of GPA33-negative CRC cell lines HT29, SW480, Caco2 and DLD1 was not sufficient to induce GPA33 protein expression in vitro (Fig. S2D).

### GPA33 expression can be induced through WNT inhibition in vitro

The inverse association of GPA33 expression and WNT signaling prompted us to investigate a possible impact of WNT on GPA33. We therefore treated SW1222 cells with the tankyrase inhibitor XAV-939 at varying densities ranging from 30% to completely confluent. We observed a significant increase in GPA33 protein and mRNA expression levels, while the effects of WNT inhibition on GPA33 induction were most prominent under medium cell confluence (Figs. 2G, H and S2E). Subsequent experiments were therefore conducted at this cell density. Similar to treatment with XAV939, siRNA-mediated or doxycycline inducible shRNA-mediated depletion of β-catenin led to an increase in GPA33 protein levels (Fig. 2I). Also, inducible expression of the dominant-negative form of TCF4 (dnTCF4) in LS174T cells had similar effects (Fig. 2I). Notably, further stimulation of SW1222 cells with Wnt3a did not increase intracellular WNT levels and also had no effect on GPA33 expression, likely due to the presence of oncogenic APC mutations (Fig. S2F). Furthermore, WNT inhibition did not induce GPA33 expression in GPA33-negative SW480 cells (Fig. S2G).

To explore the potential effects of other oncogenic signaling axes on GPA33 expression levels, we next used a panel of inhibitors to manipulate different pathway activities. However, while MAPK inhibition by AZD-6244 slightly reduced GPA33, neither inhibition of EGFR (cetuximab), TGFβ (galunisertib, SB525334), NOTCH (dibenzazepine), or modulation of BMP signaling (Noggin, BMP-2) showed significant effects on GPA33 expression levels (Fig. 2J). Due to a reduction of NOTCH activity upon WNT inhibition in these experiments, we then in more detail assessed possible effects of NOTCH inhibition on GPA33 using different culture conditions. While NOTCH activity was higher at low cell density growth, inhibitor treatment at different densities and concentrations did not significantly impact the expression levels of GPA33 antigen (Fig. S2H, I).

In summary, our experiments show that GPA33 expression levels in CRC cell lines expressing this antigen can be enhanced by inhibiting WNT activity at several levels of the signaling pathway.

### Induction of GPA33 expression in negative tumor cell populations using WNT inhibition in vivo

**Fig. 3:**
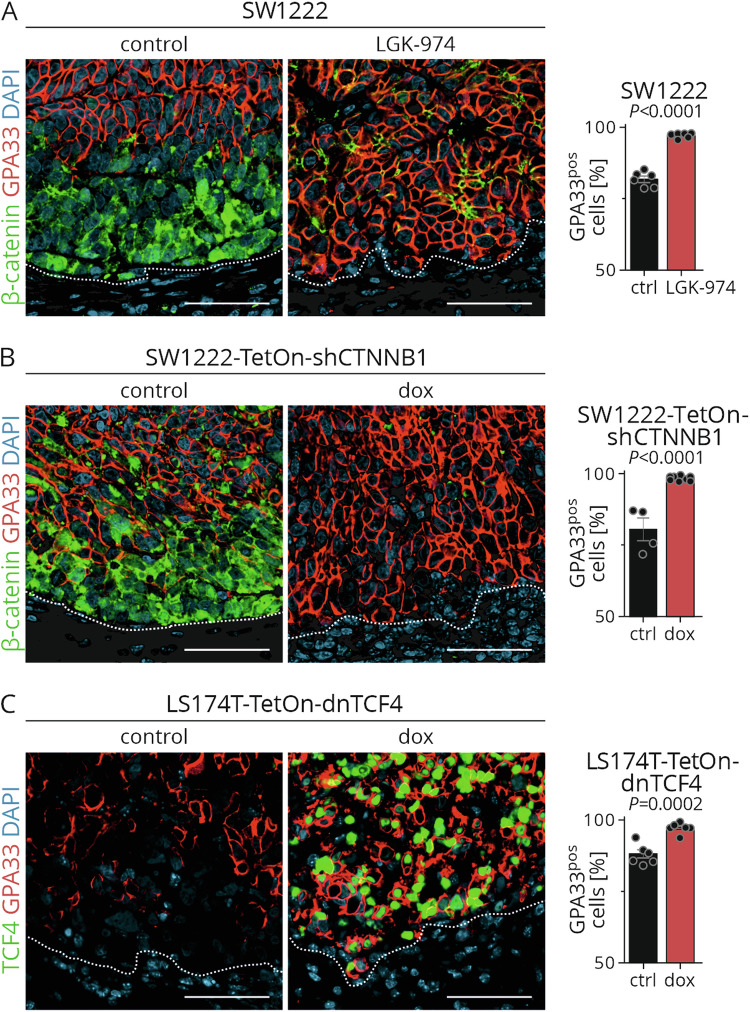
Heterogeneity of GPA33 expression can be reduced by WNT inhibition in vivo. SW1222 tumor-bearing NOD/SCID mice were treated with control or LGK-974 for 5 days (**A**); SW1222-TetOn-shCTNNB1 (**B**) or LS174T-TetOn-dnTCF4 (**C**) tumor-bearing NOD/SCID mice were treated with control or doxycycline for 4 days. Left and mid panels: Representative double immunofluorescence of xenograft tumors for indicated proteins; dotted lines represent the tumor stroma interface; scale bar: 50 µm. Right panels: Quantification of GPA33-positive cells in immunostaining of xenograft tumors; 10 fields of 250 µm^2^ were quantified for GPA33-expressing cells in each tumor; n = 6 for SW1222 and LS174T‑TetOn‑dnTCF4 tumors and n = 4 for SW1222-TetOn-shCTNNB1 controls, n = 8 for SW1222‑TetOn-shCTNNB1 doxycycline, mean ± SEM; unpaired two-tailed t-test.

To translate our findings into an in vivo model, we generated xenografts with native SW1222 colon cancer cells, and with SW1222-TetOn-shCTNNB1 or LS174T-TetOn-dnTCF4 colon cancer cells carrying doxycycline inducible WNT-repressor constructs. Untreated xenograft tumors had membranous expression of GPA33 in tumor cell populations in the tumor center that was lost towards the tumor edge (Fig. 3A–C, left panels). Also, when examining SW1222-derived xenografts for WNT activity, we observed an inverse distribution of nuclear β-catenin staining and GPA33 expression (Fig. 3A, B, left panels). These findings suggested that xenografts adequately modeled the distribution of GPA33 antigen and its inverse association with WNT that we found in primary colon cancer. In contrast, colon cancer organoids did not sufficiently reproduce GPA33 heterogeneity and thus were excluded as models for further experiments (Fig. S3).

We subsequently treated xenograft-bearing mice with the WNT inhibitor LGK974, targeting PORCN, or doxycycline to induce *shCTNNB1* or dnTCF4 expression, respectively. Upon treatment, confocal imaging showed depletion of β-catenin and strong expression of dnTCF4, respectively, indicating effective WNT repression in these xenografts (Fig. 3A–C, mid panels). Importantly, all treatments significantly increased the fraction of GPA33-positive tumor cells in this model (Fig. 3A–C, right panels). Furthermore this increase in GPA33 positive tumor cells caused reduced tumor cell heterogeneity of this antigen, resulting in an extension of GPA33 expression towards the tumor edge. These results indicate that GPA33 expression can be modulated by WNT inhibition in colon cancer in vivo resulting in almost uniform expression of this antigen in epithelial tumor cells.

### GPA33 positive colon cancer cells can be effectively targeted by CAR T cells in vitro

**Fig. 4:**
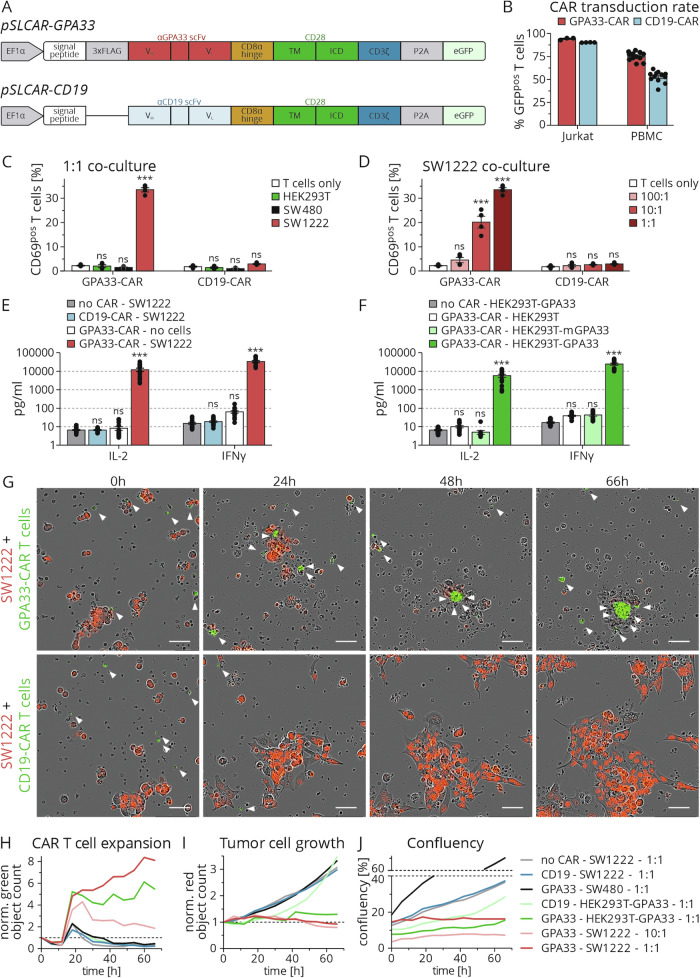
GPA33-targeted CAR T cells effectively recognize and attack GPA33-positive tumor cells in vitro. **A** Schema of the *pSLCAR-GPA33* and *pSLCAR-CD19* plasmid designs. scFv single-chain variable fragment, V_H_ variable heavy chain, V_L_ variable light chain, TM transmembrane region, ICD intracellular domain. **B** GFP expression was analyzed by flow cytometry to evaluate the CAR plasmid transduction rate in Jurkat cells or PBMCs. Transduction rate in untreated controls without co-culture is shown. n = 4 for Jurkat cells; for PBMCs, n = 4 different donors were analyzed at n = 3 replications, mean ± SEM. Percentage of CD69-positive Jurkat T cells after co-cultivation with target cells for 24 h analyzed by flow cytometry. **C** Co-culture with GPA33-negative HEK293T and SW480, or GPA33-positive SW1222 cells; **D** Co-culture with SW1222 cells at varying effector:target ratios. n = 3, mean ± SEM; 1-way ANOVA with Bonferroni multiple comparison test. **E** Untransduced, CD19-CAR or GPA33-CAR transduced PBMCs were co-cultured at a 1:1 ratio with SW1222 cells for 48 h. The IL-2 and IFNγ concentrations in the supernatant were determined using ELISA. PBMCs from 4 different donors were analyzed at n = 3, mean ± SEM; 1-way ANOVA with Bonferroni multiple comparison test. **F** Untransduced or GPA33-CAR transduced PBMCs were co-cultured with HEK293T cells with or without *pcDNA3.1-mGPA33* or *pcDNA3.1-GPA33* transfection at a 1:1 ratio for 48 h. The IL-2 and IFNγ concentrations in the supernatant were determined using ELISA. PBMCs from n = 4 different donors were analyzed at n = 3 replications, mean ± SEM; 1-way ANOVA with Bonferroni multiple comparison test. **G** Representative live cell images of CD19-CAR or GPA33-CAR transduced PBMCs during 1:1 co‑cultivation with SW1222 target cells over 66 h. CAR T cells are green and marked with white arrows; SW1222 tumor cells were stained with red nuclear dye. Scale bar: 50 µm. **H**–**J** Co-culture of CD19-CAR or GPA33-CAR transduced PBMCs with indicated target cells over 66 h. Target cells are indicated by red nuclear fluorescence. HEK293T cells were transduced with *pcDNA3.1-GPA33* to drive GPA33 expression (HEK293T-GPA33). Shown are the normalized green object counst for relative CAR T cell numbers (**H**), the normalized red object counts for relative target cell numbers (**I**) and overall confluency determined through brightfield imaging. ***P < 0.001.

In order to exploit GPA33 therapeutically, we next designed GPA33-targeting CAR T cells. We inserted a previously published GPA33 variable single-chain fragment (scFv [26]) into a CAR plasmid backbone, resulting in *pSLCAR-GPA33* (Fig. 4A). *pSLCAR-CD19* plasmid with the same layout served as control. Following lentiviral transduction, we observed vector integration in Jurkat cells as well as in human-derived PBMCs, as indicated by GFP fluorescence in flow cytometry (Fig. 4B).

To test the functionality of GPA33-CAR, we conducted a co-cultivation assay of Jurkat-derived CAR T cells with target cancer cells. Co-culture with SW1222 cells for 24 h induced the activation marker CD69 in over 30% of GPA33-CAR T cells, whereas CD19-CAR T cells remained inactive (Fig. 4C). Co-culture with the GPA33-negative cell lines HEK293T and SW480 did not result in GPA33- or CD19-CAR T cell activation (Fig. 4C). To further evaluate the sensitivity of GPA33-CAR T cells to antigen stimulation, we co-cultured them with target cells at increasing effector to target (E:T) ratios and observed a strong correlation between available antigen and T cell activation on GPA33- but not on CD19-CAR T cells (Fig. 4D).

To evaluate the effector function of CAR T cells, we assessed IL-2 and IFNγ after co-culture of PBMC-derived GPA33- and CD19-CAR T cells with or without SW1222 target cells (Fig. 4E). Co-culture of GPA33-CAR T cells led to robust secretion of both IL-2 and IFNγ in GPA33- but not in CD19-CAR T cells. IL-2 and IFNγ secretion was up to 1000-fold higher in GPA33-CAR T cells when co-cultured with SW1222 cells as compared to baseline in the absence of SW1222.

To further confirm the specific recognition of GPA33 by the GPA33-CAR, we transfected GPA33-negative HEK293T with a GPA33 expression plasmid (*pcDNA3.1-GPA33*) that resulted in high levels of GPA33 protein expression within 48 h after transfection (Fig. S4). Co-culture of GPA33-transfected HEK293T, but not of untransfected control cells caused strong IL-2 and IFNγ cytokine release, comparable to that of SW1222 co-culture (Fig. 4F). Of note, when using transgenic expression of murine mGPA33 protein, we observed no CAR T cell activation, indicating specific activation of GPA33-CAR T cells by human GPA33 (Fig. 4F).

Next, we tracked and quantified CAR T cell expansion and target cell killing in co-culture. GPA33-CAR T cells expanded already after 24 h when co-cultured with SW1222 target cells, while CD19-CAR T cells did not proliferate (Fig. 4G, H). GPA33-CAR T cells showed up to 8-fold expansion within 66 h of co-culture with SW1222 cells and 5-fold expansion after co-culture with HEK293T cells transduced with *pcDNA3.1-GPA33* (Fig. 4H). Meanwhile, tumor cell expansion, as measured by cell numbers and confluency, was effectively controlled by GPA33-CAR T cells (Fig. 4I, J). After 66 h of co-culture with GPA33-CAR T cells, we only detected few residual SW1222 tumor cells, while tumor cells challenged with CD19-CAR T cells underwent 3-fold expansion (Fig. 4G, I). On the contrary, we did not observe effects on tumor cell growth for GPA33-negative SW480 colon cancer cells challenged with GPA33-CAR T cells. These data suggest that GPA33 can be effectively and specifically targeted by CAR T cells.

### Targeting GPA33-positive colorectal cancers by CAR T cells in vivo

**Fig. 5:**
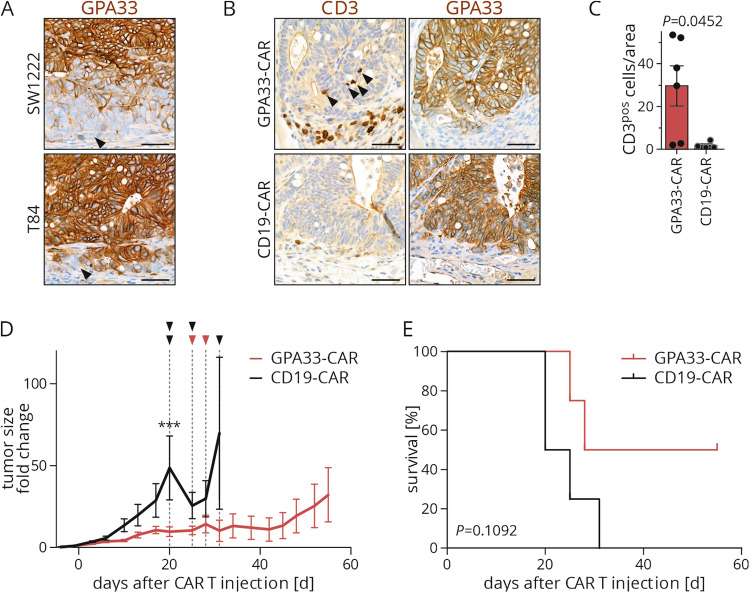
GPA33-targeted CAR T cells reduce tumor growth in T84 xenografts. **A** Representative immunostaining of GPA33 in NOD/SCID mice bearing SW1222 or T84 xenografts. Arrowheads indicate GPA33 negative tumor cells. **B**, **C** T84 tumor-bearing NOD/SCID mice received an intravenous injection of CD19- or GPA33-CAR T cells; n = 2 for CD19-CAR and n = 3 for GPA33-CAR. **B** Representative immunostaining of CD3 and GPA33; arrowheads indicate intratumoral T cells. **C** For each tumor 10 representative areas of the tumor/stroma interface were analyzed for the amount of CD3-positive cells; n = 4 for CD19-CAR and n = 6 for GPA33-CAR (2 tumors per mouse); unpaired two-tailed t-test. **D**, **E** T84 tumor-bearing NOD/SCID mice received an intratumoral injection of CD19- or GPA33-CAR T cells and tumor growth was documented until exclusion criteria were met. **D** Tumor size fold change normalized to injection day, mean ± SEM; n = 8 tumors per group (2 tumors per mouse); arrowheads and dashed lines indicate when mice had to be sacrificed. Statistical significance determined by 2-way ANOVA with Bonferroni post-tests. **E** Kaplan–Meier curve for survival of mice in indicated groups, n = 4 mice per group; log-rank test. ***P < 0.001.

To determine if GPA33-CAR T cells target GPA33-positive colon cancer in vivo, we used T84 cell line derived colon cancer xenografts. In contrast to SW1222, T84 cells form xenografts that have large GPA33 positive tumor cell populations and only few tumor cells at the tumor edge that are negative for this antigen (Fig. 5A). We treated xenograft bearing mice with a single intravenous CAR T cell injection once tumors were palpable. Analysis of these xenograft tumors 27-34 days after treatment showed a significant accumulation of human CD3 expressing GPA33-CAR T cells surrounding the tumor margin and partially infiltrating tumor epithelia (Fig. 5B, C). In contrast, xenografts where mice had been treated with CD19-CAR T cells as control only had very rare CD3 positive T cells in proximity to the tumor and did not show infiltration of the epithelial tumor component. Of note however, no significant differences in tumor growth nor changes in GPA33-antigen expression were observed with this experimental design (data not shown and Fig. 5B).

In order to further evaluate potential anti-tumor effects, we then directly injected T84 xenografts intratumorally with CAR T cells in vivo. Observation over time demonstrated slowed xenograft growth with GPA33-CAR T cells compared to CD19-CAR T cells which was significant at 20 days after injection (Fig. 5D). Of note, due to removal of xenografts reaching size dependent endpoints, significance diminished at later time points. Kaplan-Meier statistics on the same data that was based on tumor growth endpoints showed a non-significant tendency for longer survival with GPA33-CAR T cell treatments (Fig. 5E).

In summary these data suggest that GPA33-CAR T cells recognize their target antigen on xenograft tumors in vivo and may slow tumor growth.

## Discussion

GPA33 previously has been a focus for antibody-based targeted therapy due to its specific expression pattern, predominantly on gastrointestinal tissues and on the majority of CRCs [11]. While preclinical application of GPA33-targeting antibodies employing bispecific T-cell engaging antibodies [27], immunotoxins [28–30], and click-mediated radiotherapy [31] showed promising results in tumor models and xenografts, clinical trials did not provide successful response rates. A recent study combining the bi-specific T-cell engaging antibody MGD007 with PD-1 targeted immunotherapy in metastatic CRC resulted in progressive disease or death in over 50% of patients and the termination of the study (NCT03531632). Our detailed phenotypic characterization of GPA33 expressing tumors revealed a heterogeneous expression of GPA33 within colorectal cancers. GPA33 is expressed in differentiated tumor cells in the tumor center but is gradually lost towards the tumor margin, with GPA33 negative cells at the interface between tumor and tissue microenvironment. GPA33-negative cells display an undifferentiated, WNT-active stem-like phenotype, expressing EMT markers and are therefore likely to drive cancer progression upon therapy evasion. This hypothesis is further substantiated by the significant inverse association we observed between GPA33 expression levels and the presence of distant metastases or tumor progression in patient cohorts.

Cancer cells at the tumor margin interact with cells in the tumor microenvironment, such as cancer-associated fibroblasts and immune cells to establish a pro-tumorigenic niche [32]. These interactions establish a paracrine signaling loop near the tumor edge, creating a WNT-enriched niche that likely explains the observed phenotype of GPA33 negative cells in this location. In line with this, we demonstrate that inhibition of WNT signaling in vivo induced GPA33 expression in tumor cells at the tumor edge and reduced intratumoral heterogeneity for this antigen. Importantly, we observed a consistent phenotype of GPA33 expression patterns of primary tumors and their distant metastases. This suggests that GPA33 expression reflects CRC cell plasticity. GPA33-negative stem-like cancer cells may undergo EMT and disseminate to distant organs where GPA33 expression is regained at the metastatic site, likely due to differentiation gradients established by microenvironmental cues. This consistency suggests that primary tumor biopsies may be used to determine patient eligibility for GPA33-targeted therapies aiming at both the primary tumor and its metastatic lesions.

Application of WNT inhibitors in vitro and in vivo identified GPA33 as a target of this pathway. The detailed regulatory mechanisms of GPA33 gene expression are, however, complex and conflicting data have been reported. Contrary to previous research [25, 33], we observed that GPA33 expression is regulated by CDX1, but not by KLF4. CDX1, a known driver of intestine-specific gene expression [34] and a tumor suppressor [35], is frequently silenced by promotor methylation in CRC [36]. It has been reported as a WNT target gene during embryonic development [37] and in adults it is predominantly expressed in intestinal crypts [38]. These observations contradict the inverse correlation of CDX1 expression with WNT activity we observed and indicate a context-specific function of CDX1. Furthermore, a cooperative role of CDX1 and EHF, two epithelial specific transcription factors, in maintaining differentiation of cells of the enterocyte lineage was recently described [39]. GPA33 expression in a GPA33-negative cell line was only achieved by simultaneous transfection of plasmids encoding both transcription factors suggesting a more complex interplay between CDX1 and EHF in GPA33 regulation.

GPA33-negative cells marked a specific tumor cell subset that is characterized by high WNT activation. However, WNT signaling also is responsible for the maintenance of stem cell compartments outside the gastrointestinal tract [40]. Therefore WNT inhibitors may have side effects in tissues that harbor stem cell-like populations and undergo constant self-renewal. Despite these challenges, several targeting strategies are being evaluated in clinical trials, including targeting β-catenin and its transcriptional partners, the APC destruction complex, WNT receptors, or WNT ligands [41]. A phase I clinical trial for the PORCN inhibitor LGK-974 demonstrated effective WNT inhibition in solid tumors and sufficient drug tolerability in patients [42]. We show that treatment of tumor-bearing mice with LGK-974 resulted in nearly all cancer cells expressing GPA33. This suggests that inhibition of WNT in human patients to enhance GPA33 expression in colon cancer may be feasible for induction of this therapeutic target.

As an alternative to the yet clinically unsuccessful antibody-based targeted therapy, we established CAR T cells against GPA33. Due to their cellular nature, CAR T cells can expand and differentiate into multiple T cell effector types for an enhanced sensitivity and longer-lasting anti-tumor effects [43]. However, CAR T cell therapy of solid tumors remains challenging due to insufficient tumor tissue infiltration, CAR T cell exhaustion, the immunosuppressive microenvironment and off-tumor toxicities in healthy tissue [44]. Albeit strong CAR T cell activation in vitro and high GPA33 antigen expression in T84-derived xenografts, we observed only limited effects on tumor growth and low CAR T cell infiltration of the tumor in vivo, which may be related to T cell exhaustion. However, we found a significant anti-tumor response after intratumoral GPA33-CAR T cell injection into colon cancer xenografts. Local administration of CAR T cells has recently been demonstrated to increase anti-tumor response and a localized immune activation in clinical trials with solid tumors [45–47]. The local administration of CAR T cells to CRC metastases may therefore have a positive effect on CAR T cell invasion and potentially limit adverse events related to toxicity in healthy tissue. Nevertheless, our study only provides initial proof of concept that GPA33-directed CAR T cell immunotherapy may be feasible. Further independent in vivo studies that in detail address efficacy and potential side effects of this approach are necessary as a basis for future clinical trials.

Concerns for therapeutic side effects arise from high expression of GPA33 in healthy gastrointestinal epithelia. Furthermore, GPA33 has recently been identified as a marker for naïve regulatory T cells [48], while the mechanisms driving GPA33 expression in these immune cells are still unknown. This poses an additional challenge for off-target toxicity concerns as treatment with GPA33-CAR T cells may lead to damage of healthy intestinal epithelia or abrogation of certain T cell species. Given that our CAR is specific to human GPA33, our xenograft model did not allow the assessment of such potential off-target effects. Further study will thus be necessary to determine if GPA33 directed CAR T cells may be cancer specific. Examples of CAR T cells that spare healthy tissues due to antigen shielding have been published for other intestinal antigens, such as CEA and CDH17 [49, 50].

A further important aspect of a potential GPA33 CAR T therapy is the role of active WNT signaling in the tumor microenvironment [51]. TCF1, a target of canonical WNT signaling, negatively modulates FOXP3 transcriptional activity in immunosuppressive regulatory T cells [52]. Active WNT signaling also halts T cell differentiation and drives the generation of T memory stem cells [53]. Combining CAR T cell therapy against CRC with WNT inhibition has been suggested to enhance tumor infiltration [54]. Our data provide the rationale to investigate combinations of CAR T cell therapy with WNT inhibition that may not only have beneficial effects on reduced GPA33 heterogeneity but could also positively promote CAR T effectiveness.

While our study demonstrates a functionally improved approach for epitope-targeted therapies, it also highlights the importance of analyzing intratumoral heterogeneity and the underlying regulatory framework of target gene expression. The failure of previous anti-GPA33 targeted therapies in clinical trials may have been caused by GPA33-negative stem-like tumor cells or the inclusion of patients with low overall GPA33 expression levels. Heterogeneous intratumoral expression in CRC has been demonstrated for several antigens, including PD-L1 [55] and CEA [56]. CEA, like GPA33, showed WNT-dependent intratumoral heterogeneity in an in vitro study using patient-derived organoids [56]. CEA-targeted CAR T-cell therapy is currently being investigated in more than 10 phase I and I/II clinical trials for CRC [57]. Because of the WNT-dependent regulation of CEA, this antigen may be another candidate for combined immunotherapy and WNT inhibitors. Thus, re-evaluation of known tumor antigens in the context of tumor heterogeneity may reveal novel powerful drug-immune combinations to enhance immunotherapeutic responses. In addition, patients receiving targeted immunotherapy might benefit from the evaluation of intratumoral distributions of target antigens prior to the initiation of treatment.

## Materials and methods

### TCGA data and clinical samples

TCGA-COAD data and GPA33 RNA expression levels were from The Human Protein Atlas, comprising 438 patients across various UICC stages and included information on microsatellite status. CRC specimens were from patients who underwent surgical resection at the University of Munich (LMU, Munich, Germany) between 1994 and 2007, and obtained from the archives of the Institute of Pathology. Follow-up data were recorded prospectively by the Munich Cancer Registry. Specimens were anonymized, and the need for consent was waived by the institutional ethics committee of the Medical Faculty of the LMU. The metastasis collection (M0M1) included 64 patients in a case-control design and sample size was based on previous experience and limited by the availability of tumor tissue. Half of the patients had colorectal cancer with synchronous liver metastasis. The control group consisted of patients with colorectal cancer who had no distant metastases at diagnosis and a disease-free survival of at least 5 years after primary surgical resection. Cases and controls were matched by tumor grade, T category, and tumor location. 8 tumor specimens were mismatch repair deficient (dMMR) due to loss of MLH1 expression. The UICC stage II collection consisted of colorectal adenocarcinomas with bowel wall infiltration (T3 and T4), but without nodal (N0) or distant metastases (M0) at diagnosis and included 225 patients. At a power of 0.8 with α = 0.05, relative risks of 2.2 for cancer specific survival and 1.9 for disease free survival could be detected in this collection. Two samples were excluded due to insufficient tumor material. Fourteen samples were selected for immunofluorescence staining based on GPA33 expression levels and gradients. Optimal expression cut-offs were determined by ROC curves. The cut-off value for the TCGA-COAD data was 54.3 fragments per kilobase per million mapped reads (FPKM) *GPA33* mRNA, 85.2% GPA33 positive cells for the UICC II collection, and 84.6% positive cells for the M0M1 collection. Survival status and time since the last follow-up were plotted against GPA33-levels using Prism (GraphPad).

### Cell culture and in vitro treatments

Caco2, Cal29, Colo205, DLD1, HEK293T, HT29, Jurkat, LS174T, SW403, SW480, SW948, and T84 cells were obtained from ATCC. SW1222 cells were a gift from the Ludwig Institute for Cancer Research (New York, NY, USA). LS174T-TetOn-dnTCF4 cells were a gift from Hans Clevers [58]. All cell lines were cultured at 37 °C with 5% CO_2_/20% O_2_, underwent short tandem repeat (STR) cell line authentication prior to use and tested negative for mycoplasma contamination. Jurkat cells were cultured in RPMI-1640 (Sigma) and SW948 in Leibovitz’s L15 (Lonza) each supplemented with 10% FBS (Merck or PAN Biotech) and 100 U/mL penicillin–streptomycin (Biochrom). All remaining cell lines were cultured in DMEM (Sigma) supplemented with 2 mM ultraglutamine (Lonza), 10% FBS (Merck or PAN Biotech), and 100 U/mL penicillin-streptomycin (Biochrom). For perturbation assays cells were seeded and treated for 72 h with 10 µM XAV-939 (Selleckchem; in DMSO), 0.5 µM AZD-6244 (Selleckchem; in DMSO), 10 µg/mL Cetuximab (Merck Serono; Erbitux), 10 µM Galunisertib (Selleckchem; in DMSO), 1 µM SB25334 (Selleckchem; in DMSO), 5–20 µM DBZ (Selleckchem; in DMSO), 250 ng/mL Noggin (Peprotech; in PBS) or 200 ng/mL BMP-2 (R&D; in 4 mM HCl). For WNT activation assays cells were seeded and treated for 48 h with 100 ng/mL human Wnt3a (hWnt3a; R&D systems; in PBS + 0.1% BSA), 100 ng/mL murine Wnt3a (mWnt3a; Time Bioscience; in PBS + 0.1% BSA), or with 100 ng/mL Wnt3a-conditioned medium. For transgene activation in LS174T-TetOn-dnTCF4 and SW1222-TetOn-shCTNNB1 cells, cells were treated with 2 µg/mL doxycycline (Sigma; in H_2_O) for 48 h. For siRNA knockdown of target genes, cells were transfected with Lipofectamine RNAiMAX (Invitrogen) according to the manufacturer’s instructions. Harvest and confirmation of knockdown followed after incubation for 72 h.

### Tumor xenografts and in vivo treatments

Female NOD/SCID mice (NOD.CB17*-Prkdc*^*scid/scid*^, Janvier Labs) were housed under pathogen-free conditions on a 12-hour day/night cycle with free access to food and water, according to EU regulations. All experiments were reviewed and approved by the Landesamt für Gesundheit und Soziales Berlin, according to the Berlin State guidelines. Sample sizes were based on preliminary data and previous experience. T84, SW1222, SW1222-TetOn-shCTNNB1, or LS174T-TetOn-dnTCF4 cells were suspended in 200 µL of a 1:1 mixture of DMEM and growth factor-depleted Matrigel (Corning) and injected subcutaneously in both flanks of 8-10-wk-old mice for xenograft formation. For WNT inhibition assays, mice were randomly assigned to control or treatment groups when tumor diameters reached 1 cm. SW1222-bearing mice were treated *p.o.* daily for 5 days with 5 mg/kg/day LGK-974 (Selleckchem S7143) in citrate buffer (10% v/v citrate buffer pH 2.5 + 90% (v/v) citrate buffer pH 3.0) and sacrificed on day 6. SW1222-TetOn-shCTNNB1-bearing mice were treated *i.p.* every other day for 4 days (days 1, 3, 5, and 7) with 4 mg/kg/day doxycycline (Sigma) in PBS and sacrificed on day 8. LS174T-TetOn-dnTCF4-bearing mice were treated *p.o.* daily for 4 days with 1 mg/day doxycycline (Sigma) in H_2_O and sacrificed on day 4. For adoptive transfer of CAR T cells, mice received a singular intravenous injection of 10 × 10^6^ CAR T cells (adjusted to transduction rate) in 200 µL PBS or an intratumoral injection of 2.5 × 10^6^ CAR T cells (adjusted to transduction rate) in 50 µL PBS in each tumor as soon as they were palpable. Tumor size was measured twice weekly, and animals were sacrificed once the tumor size reached a diameter of 1.5 cm. No xenografts were excluded from analyses. Investigators were not blinded to group allocations.

### Functional CAR T cell assays

To measure CD69 levels on CAR T cells, 10.000 transduced Jurkat cells were co-cultivated with SW1222, SW480 or HEK293T target cells at 1:1, 1:0.1 or 1:0.01 effector:target ratio for 24 h. Jurkat activation was assessed by co-expression of GFP and CD69 using an anti-human PE-conjugated CD69 antibody (Invitrogen), as previously described [59]. The CD69-PE antibody was diluted 1:250 in 50 µL of PBS per well and added directly to the cells for 30 min at 37 °C. The cells were fixed with PFA at a final concentration of 0.5% and analyzed on the CytoFLEX S (Beckman Coulter). For cytokine release assays, 30.000 transduced PBMCs were co-cultivated with target cells at 1:1 or 1:0.1 effector:target ratio for 48 h in TCM. Supernatant was collected and analyzed for IL-2 and IFNγ concentrations using the OptEIA ELISA kits (both BD Biosciences) according to the manufacturer’s instructions. Experiments were confirmed with PBMCs from four different donors. To visualize target cells in live cell imaging, SW1222, HEK293T and SW480 cells were transduced with commercially obtained lentivirus (Lenti Nuclight Red, Incucyte Sartorius). 10.000 transduced PBMCs were cultivated with target cells at 1:1 or 1:0.1 effector:target ratio for 66 h in TCM. Transduced PBMCs were added five hours after seeding target cells. GFP of CAR T cells and mKate2 signal of the target cells were documented in an Incucyte S3 instrument (Sartorius) for 66 h every six hours. Experiments were confirmed with PBMCs from two different donors.

### Statistical analysis

Appropriate statistical tests were used to compare data with similar variances and are referenced in figure legends. Biological replicates are given as n values. All graphs show mean and error bars represent standard error of the mean (SEM). Differences were considered statistically significant when *P* < 0.05 and *P* values are given within figures or figure legends. Statistical significance was determined with the GraphPad Prism software.

### Other methods

Other methods are included in the Supplementary information.

## Supplementary information

Supplementary information

## Data Availability

TCGA-COAD data and GPA33 RNA expression levels were sourced from The Human Protein Atlas. The scRNA-seq dataset analyzed in this study is available in the GEO database: GEO GSE166555. All other datasets generated and/or analyzed during the current study are available from the corresponding author on reasonable request.
