# Supplementary material for: GPA33 expression in colorectal cancer can be induced by WNT inhibition and targeted by cellular therapy
Source: Oncogene. 2024 Oct 29;44(1):30–41. doi: 10.1038/s41388-024-03200-3 (PMC11700846; doi:10.1038/s41388-024-03200-3)
Supplement: Supplementary file 1 — Supplementary information [file 41388_2024_3200_MOESM1_ESM.pdf]

Supplementary information

GPA33 expression in colorectal cancer can be induced by WNT inhibition and targeted by cellular therapy

Teresa Börding, Tobias Janik, Philip Bischoff, Markus Morkel, Christine Sers, David Horst

Supplementary Figures Page 2

Supplementary Tables Page 7

Supplementary Methods Page 10

Supplementary References Page 13

Supplementary Figures

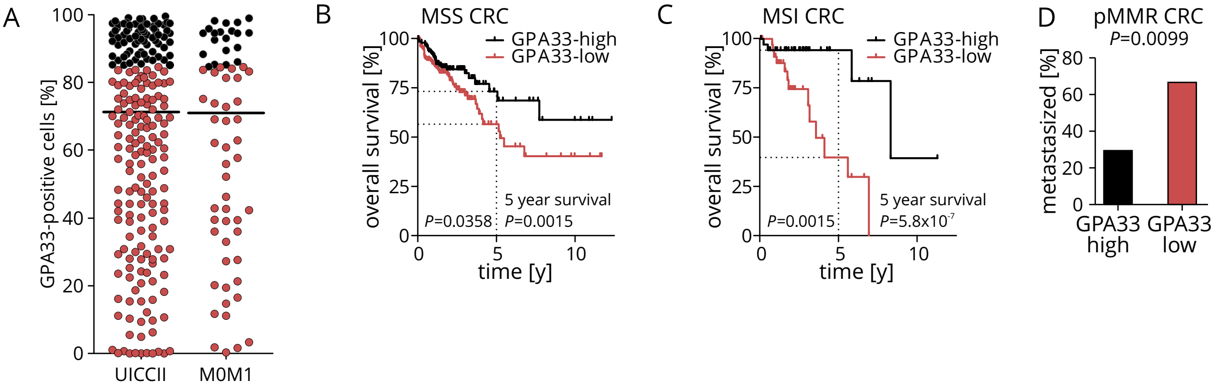

Supplementary Figure S1:

(A) Quantification of GPA33-positive cells in immunostaining for each case in the collection of UICCII patients (UICCII) and matched case-control collection (M0M1). The straight line represents the median for each collection; red represents cases of low GPA33 and black represents cases of high GPA33 expression.

(B-C) Kaplan-Meier curve for overall survival of (B) MSS and (C) MSI CRC patients with GPA33-high and GPA33-low RNA expression levels in the TCGA-COAD cohort; log-rank test for overall survival and chi-square test for 5-year survival.

(D) Association between GPA33 protein expression levels and liver metastasis in a matched case-control collection of 56 pMMR colon cancers; chi-square test.

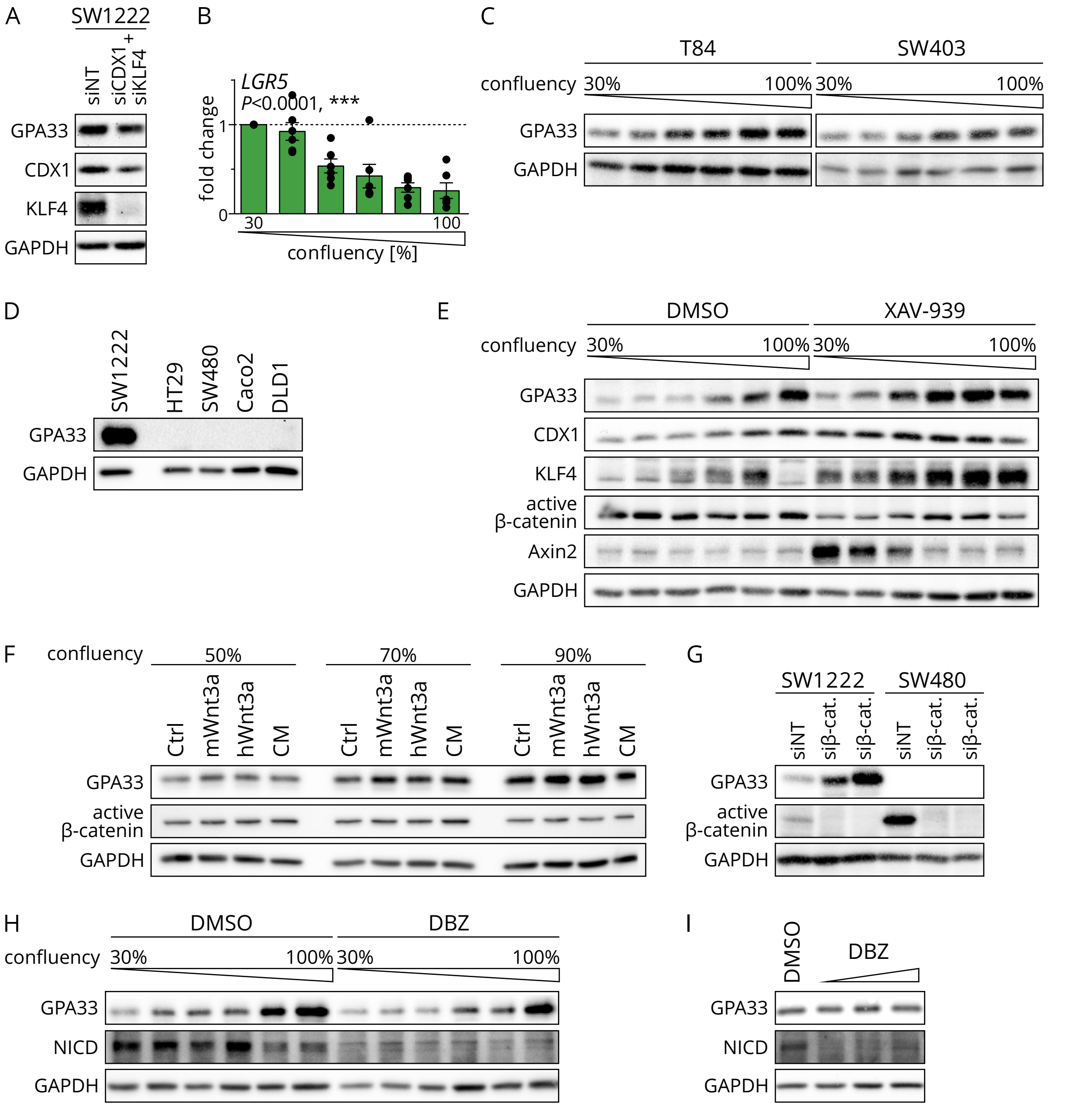

Supplementary Figure S2:

(A) Representative immunoblots for the indicated antigens of SW1222 cells treated with control (siNT) or siCDX1 and siKLF4 for 72h.

(B) LGR5 mRNA levels at varying cell confluencies. SW1222 cells were treated with DMSO for 72h. Normalized to lowest cell confluency. n=6, 1-way ANOVA. *, $P<0.05$; **, $P<0.01$; ***, $P<0.001$

(C) T84 or SW403 cells were treated with DMSO for 72h. Representative immunoblots for the indicated antigens and cell confluency.

Figure legend continued on next page.

Supplementary Figure S2: *(continued)*

(D) HT29, SW480, Caco2 and DLD1 cells were harvested at 100% cell confluency and SW1222 at 50% cell confluency after treatment with DMSO for 72h. Representative immunoblots for the indicated antigens.

(E) SW1222 cells were treated with the control (DMSO) or 10 µM XAV-939 for 72h and harvested at varying cell confluencies. Representative immunoblots for the indicated antigens, treatments, and confluencies.

(F) SW1222 cells were treated with control (PBS + 0.1% BSA), 100 ng/mL murine Wnt3a (mWnt3a), 100 ng/mL human Wnt3a (hWnt3a) or 100 ng/mL WNT conditioned medium (CM) for 48h and harvested at varying cell confluencies. Representative immunoblots for the indicated antigens, treatments and confluencies.

(G) The indicated cell lines were treated with control (siNT) or siβ-catenin for 72h and harvested at 50% cell confluency. Representative immunoblot for the indicated antigens.

(H) SW1222 cells were treated with control (DMSO) or 10 µM DBZ for 72h and harvested at varying confluencies. Representative immunoblots for the indicated antigens, treatments and confluencies.

(I) SW1222 cells were treated with control (DMSO), 5 µM DBZ, 10 µM DBZ or 20 µM DBZ for 72h and harvested at 50% confluency. Representative immunoblots for the indicated antigens, treatments and confluencies.

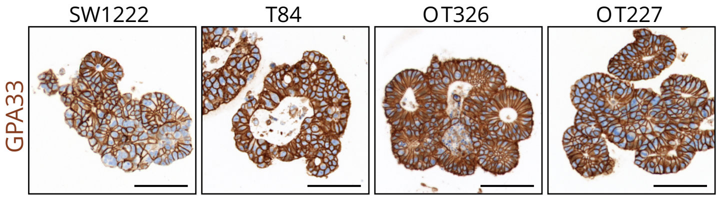

Supplementary Figure S3:

Representative GPA33 immunostaining of paraffin embedded CRC organoids derived from SW1222 and T84 CRC cell lines or patient-derived OT326 and OT227 organoid lines. Scale bars: 100 μM.

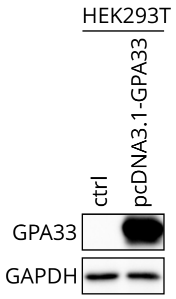

Supplementary Figure S4:

HEK293T cells were transfected with control (*pcDNA3.1*) or *pcDNA3.1-GPA33* and harvested after 48h. Representative immunoblot of protein lysates for the indicated antigens.

Supplementary Tables

Supplementary Table S1: Association of GPA33 expression with clinical outcome in collection of stage II colon carcinomas with documented follow-up.

Column percent values for totals and row percent values for GPA33 categories are given in parentheses; chi-square test.

Characteristics	Total	GPA33 expression		*P*
		low	high	
All patients	223 (100)	150 (67.3)	73 (32.7)	
Age (y, median 69.3)				
≤ 69	112 (50.2)	77 (68.8)	35 (31.2)	0.63
≥ 70	111 (49.8)	73 (65.8)	38 (34.2)	
Sex				
Male	120 (53.8)	80 (66.6)	40 (33.3)	0.84
Female	103 (46.2)	70 (68.0)	33 (32.0)	
T-stage (UICC)				
T3	184 (82.5)	119 (64.7)	65 (35.3)	0.06
T4	39 (17.5)	31 (79.5)	8 (20.5)	
KRASmut				
Yes	84 (38.0)	54 (64.2)	32 (35.7)	0.35
No	137 (62.0)	95 (69.3)	43 (30.7)	

Supplementary Table S2: Association of GPA33 expression with clinical outcome in matched case-control collection of metastatic and non-metastatic colon carcinomas.

Column percent values for totals and row percent values for GPA33 categories are given in parentheses; chi-square test.

**Characteristics**	**Total**	**GPA33 expression**		***P***
		**low**	**high**	
All patients	64 (100)	44 (68.7)	20 (31.3)	
Age (y, median 69.0)				
≤ 69	33 (51.6)	24 (72.7)	9 (27.2)	0.48
≥ 70	31 (48.4)	20 (64.5)	11 (35.5)	
Sex				
Male	29 (45.3)	17 (58.6)	12 (41.4)	0.11
Female	35 (54.7)	27 (77.1)	8 (22.9)	
T-stage (UICC)				
T3	51 (79.7)	35 (68.6)	16 (31.4)	0.97
T4	13 (20.3)	9 (69.2)	4 (30.8)	
Metastasis				
Yes	32 (50.0)	26 (81.2)	6 (18.8)	0.03
No	32 (50.0)	18 (56.2)	14 (43.8)	
Grade				
Low	16 (25.0)	8 (50.0)	8 (50.0)	0.06
High	48 (75.0)	36 (75.0)	12 (25.0)	

Supplementary Table S3: Primer sets used for quantitative real-time PCR.

Gene	Forward primer	Reverse primer
GPA33	ACTTCCACCTCCAGTCGAGA	AATGGTGATGGAGGCATCGG
CDX1	GGGATAAGGGAGTCCAGGGT	CCTCAGGTCATGCTCTGCAA
KLF4	CGTTGACTTTGGGGTTCAGG	AAGTCGCTTCATGTGGGAGA
LGR5	ACCTGAAAGCCCTTCATTCA	TGCTATGGTCCACACTCCAA
UBE2D2	CAGTAATGGCAGCATTTGTCTTGA	TCATCTGGATTGGGATCACACA

Supplementary Methods

Histology and immunostaining

For immunohistochemistry (IHC) or immunofluorescence (IF), 3 µm sections of colorectal cancer, tissue microarray (TMA), or xenograft samples were deparaffinized and stained on a DISCOVERY ULTRA autostainer (Ventana Medical Systems). Primary antibodies were directed against GPA33 (abcam, ab108938, 1:500 or Atlas, HPA018858, 1:300), β-catenin (Cell Signaling, #9587, 1:50), LAMC2 (Millipore, MAB19562, 1:50), E-cadherin (Cell Signaling, #14472, 1:200), Ki67 (Cell Signaling, #9027, 1:400), TCF4 (Santa Cruz, sc-166699, 1:100) or CD3 (Agilent, A0452, 1:100). Secondary antibodies for IF were coupled to either FAM, Cy5, or Red610 fluorophores using Roche Discovery kits (FAM: 7988150001, Cy5: 7551215001, Red610: 7988176001, Roche Ventana). DAPI (Invitrogen, 0.1 µg/mL) was used for nuclear staining. IHC staining was visualized using an ultraView or OptiView DAB detection kit (Ventana Medical Systems). IHC slides were scanned using a Panoramic SCAN 150 scanner (3DHISTECH) and quantified in QuPath (QuPath). GPA33-positive cells were detected using the positive cell detection protocol in the Cell: DAB OD mean channel with a threshold of > 0.05. To visualize and quantify the amplification of GPA33 expression levels in xenografts, the positive cell detection protocol was run using multiple thresholds (threshold 1+: 0.05; threshold 2+: 0.3, threshold 3+: 0.6). CD3-positive cells were quantified by choosing 10 square (250x250 µm) representative areas per tumor and counting CD3-positive cells. Each square was located at the tumor edge and contained 50 % tumor and 50 % stroma. Confocal IF images were taken on Yokogawa Cell Voyager CQ1 confocal microscope (Yokogawa). Contrast and brightness were adjusted using Photoshop (Adobe). Co-expression was quantified using QuPath (QuPath). Representative tumor areas at the tumor edge and tumor center were selected and the cell detection protocol was run in all channels. The mean relative fluorescence intensity (% RFI) was calculated in relation to the highest score within each channel in each analyzed TMA core. β-catenin and Ki67 nuclear staining were quantified, whereas for GPA33, LAMC2 and E-cadherin staining of the entire cell was considered. The top 25% of GPA33 expressing cells were considered as GPA33-high and the bottom 25 % of GPA33 expressing cells were considered as GPA33-low.

Single cell RNA sequencing

A previously described single-cell RNA sequencing (scRNAseq) dataset [1] was analyzed using the Seurat (v4.4.0, [2]) and PROGENy (v1.24.0, [3]) packages. An intregrated UMAP was created of the dataset filtered for copy-number-aberrant cells. Tumors with fewer than 100 GPA33-positive cells (>1 read for GPA33) were excluded from the analysis, leaving four tumor samples. For analysis of signaling pathways and differentially expressed genes, cells were subset into GPA33-high and GPA33-low groups based on average expression levels, with the GPA33-high group assigned to average expression above 1. PROGENy scores were calculated to quantify signaling pathway activity. The FindAllMarkers function was used to identify DEGs for each subset, setting mind.diff.pct to 0.169.

Immunoblotting

For immunoblotting, freshly harvested cells were resuspended in M-PER buffer (Thermo Fisher Scientific) containing freshly added protease and phosphatase inhibitors (both Roche). Samples were

sonicated for 5 min, centrifuged, and protein concentration was determined using a Pierce BCA Protein Assay Kit (Thermo Fisher Scientific). Equal amounts of protein lysate were mixed with sample buffer and heated for 5 min at 95°C. Proteins were separated by SDS-PAGE and transferred to a PVDF membrane (Merck) using a semi-dry transfer. Membranes were incubated with primary antibodies directed against GPA33 (abcam, ab108938, 1:10.000), active β-catenin (anti ABC) (Millipore, 05-665, 1:1000), CDX1 (abcam, ab126748, 1:10.000), KLF4 (Atlas, HPA002926, 1:500), GAPDH (Ambion, AM4300, 1:400.000), Axin2 (Cell Signaling, #2151, 1:1000), pERK (NEB, #9101, 1:750), totERK (Cell Signaling, #9107, 1:750), pSMAD2 (Cell Signaling, #3108, 1:1000), NICD (Cell Signaling, #4147, 1:500) or pSMAD1/5/8 (NEB, #9511, 1:1000). Bands were visualized using HRP-conjugated secondary mouse (Cell Signaling, #7076, 1:1000) or rabbit (Cell Signaling, #7074, 1:1000) antibodies and Immobilon Western Chemiluminescent HRP Substrate (Millipore).

Gene expression analysis

RNA was isolated using the RNeasy Mini Kit (Qiagen) according to the manufacturer's instructions. cDNA was prepared of 500 ng isolated RNA using the Quantitect Reverse Transcription Kit (Qiagen), and quantitative real-time PCR was performed using standard protocols for GoTaq qPCR Master Mix (Promega) using the primers listed in Table S1. Relative expression was calculated using the ΔΔCt method with UBE2D2 for normalization.

Cloning

The sequence for shCTNNB1 (clone ID TRCN0000003845) was obtained from the Genetic Perturbation Platform (Broad Institute) and cloned into the Tet-pLKO-puro backbone (Addgene #21915) using AgeI and EcoRI restriction sites, as described previously [4]. The sequence for the anti-GPA33 short-chain variable fragment (scFv) has been previously published [5]. GPA33 scFv clone B was cloned into the *pSLCAR* backbone (Addgene #135991) using BpiI restriction sites according to a previously published protocol [6]. A 3xFLAG-tag and CD8α hinge were added to our insert (IDT-DNA). The coding sequences for human (RefSeq NM_005814.3) and murine (RefSeq NM_021610.2) *GPA33* mRNA were obtained from the National Library of Medicine (NCBI). BamHI and XbaI restriction sites were added (IDT-DNA) and inserts were cloned into the *pcDNA3.1* backbone resulting in *pcDNA3.1-GPA33* and *pcDNA3.1-mGpa33* expression plasmids, respectively. For transduction, lentivirus was produced in HEK293T cells by co-transfection of 8.4 µg plasmid with 6 µg psPAX2 (Addgene #12260) and 3.6 µg pMD2.G (Addgene #12259) lentiviral vectors. The virus-containing medium was passed through 0.45 µm filters (Sarstedt). For transduction of SW1222 and Jurkat cells, the viral supernatant was mixed 1:1 with culture medium and 8 µg/mL polybrene (Santa Cruz), and added to the cells for 24h. Successful integration was tested using puromycin selection or flow cytometry to detect GFP signals. For SW1222-TetOn-shCTNNB1, single-cell clones were sorted in 96-well plates and expanded under puromycin selection (2 µg/mL, Sigma). For transient transfections Lipofectamine 3000 (Invitrogen) was used according to manufacturer's protocol. Confirmation of transfection followed after incubation for 24h-72h.

Wnt3a-conditioned medium

Wnt3a-conditioned medium was produced and tested to have an activity equivalent to 100 ng/mL Wnt3a using a previously described protocol [7]. Briefly, Wnt3a-producing L cells were cultured and the

resulting Wnt3a-containing culture medium was harvested, filtered and stored at -20°C. Activity was tested using a TOP/FOP luciferase reporter assay.

Organoid culture & embedding

The patient-derived organoid lines OT227 and OT326 were obtained from the OncoTrack biobank [8]. For 3D organoid culture T84 and SW1222 cell lines or organoid lines were cultured in matrigel (Corning) with organoid medium containing Advanced DMEM/F12 (Gibco), 1x N-2 supplement (Gibco), 1x B-27 supplement (Gibco), 1x GlutaMAX supplement (Gibco), penicillin-streptomycin (Biochrom), 10 mM HEPES (Corning), 1 mM N-Acetyl-L-cysteine (Sigma-Aldrich), 50 ng/ml EGF (PeproTech), 20 ng/ml FGF (Sigma-Aldrich). After growth to a sufficient size, organoids were pelleted, resuspended in 200 µL histogel and paraffin embedded.

Human T cell transduction

Human peripheral blood mononuclear cells (PBMCs) were isolated from whole blood obtained from healthy donors by density gradient centrifugation using BioColl cell separating solution (Merck). All blood donations were made with informed consent and with the approval of the Charité ethics committee (EA1/196/22). PBMCs were cultured in T cell medium (TCM) containing RPMI (Gibco), 10 % FBS (Merck or PAN Biotech), 1 mM sodium pyruvate (Gibco), 0.1 mM MEM non-essential amino acids (Gibco), and 100 U/ml penicillin-streptomycin (Biochrom). TCM was supplemented with either IL-2 (10 ng/mL, Peprotech) or IL-7/IL-15 (10 ng/mL each, both Miltenyi) if the cells were used for *in vitro* or *in vivo* assays, respectively. For CAR plasmid transduction, we followed a previously published protocol [9]. PBMCs were seeded into 24-well plates coated with anti-human CD3 (5 µg/µL, BioLegend) and anti-human CD28 (1 µg/µL, BioLegend) for 48h. Meanwhile, virus-coated 24-well plates were prepared: plates were blocked with 500 µL 2 % BSA and PBS per well for 30 min at 37°C, washed twice with PBS, and stored at 4°C overnight. The PBS was then exchanged with 500 µL of lentivirus-containing supernatant and centrifuged (3000 × g, 2h, 4°C). Afterwards, the supernatant was replaced with 1 mL activated PBMCs, 1 mL lentiviral supernatant and either IL-2 or IL-7/IL-15. The plates were centrifuged (800 × g, 30 min, 32°C). 1 mL of supernatant was replaced with 1 mL TCM and interleukins for overnight culture. The next day, 1 mL was replaced with lentiviral supernatant, and the centrifugation was repeated (800 × g, 30 min, 32°C), and the cells were expanded. For IL-2 cultures, TCM with IL-2 was added every couple of days, whenever the medium turned yellow. After 10 days, cells were frozen in liquid nitrogen until *in vitro* use. For IL-7/IL-15 cultures, when the medium turned yellow, some medium was removed, and a double amount was added. After 7-12 days, cells were washed twice in PBS and passed through a 40 µm cell strainer (Falcon) before the transduction rate was determined using flow cytometry and CAR T cells were used for *in vivo* assays directly. To determine transduction efficiency of T cells, cell suspension was fixed with PFA at a final concentration of 0.5 % and measured directly on the CytoFLEX S (Beckman Coulter) for GFP signal.

Supplementary References

1. Uhlitz F, Bischoff P, Peidli S, Sieber A, Trinks A, L€ Uthen M, et al. Mitogen-activated protein kinase activity drives cell trajectories in colorectal cancer. EMBO Mol Med. 2021 Oct 7;13(10):e14123.

2. Hao Y, Hao S, Andersen-Nissen E, Mauck WM, Zheng S, Butler A, et al. Integrated analysis of multimodal single-cell data. Cell. 2021 Jun 24;184(13):3573-3587.e29.

3. Schubert M, Klinger B, Klünemann M, Sieber A, Uhlitz F, Sauer S, et al. Perturbation-response genes reveal signaling footprints in cancer gene expression. Nat Commun 2017 91. 2018 Jan 2;9(1):1–11.

4. Wiederschain D, Wee S, Chen L, Loo A, Yang G, Huang A, et al. Single-vector inducible lentiviral RNAi system for oncology target validation. Cell Cycle. 2009 Feb 1;8(3):498–504.

5. Rader C, Ritter G, Nathan S, Elia M, Gout I, Jungbluth AA, et al. The Rabbit Antibody Repertoire as a Novel Source for the Generation of Therapeutic Human Antibodies. J Biol Chem. 2000 May 5;275(18):13668–76.

6. Bloemberg D, Nguyen T, MacLean S, Zafer A, Gadoury C, Gurnani K, et al. A High-Throughput Method for Characterizing Novel Chimeric Antigen Receptors in Jurkat Cells. Mol Ther - Methods Clin Dev. 2020 Mar 13;16:238–54.

7. Wallaschek N, Niklas C, Pompaiah M, Wiegering A, Germer CT, Kircher S, et al. Establishing Pure Cancer Organoid Cultures: Identification, Selection and Verification of Cancer Phenotypes and Genotypes. J Mol Biol. 2019 Jul 12;431(15):2884–93.

8. Schütte M, Risch T, Abdavi-Azar N, Boehnke K, Schumacher D, Keil M, et al. Molecular dissection of colorectal cancer in pre-clinical models identifies biomarkers predicting sensitivity to EGFR inhibitors. Nat Commun. 2017 Apr 10;8(1):14262.

9. Bunse M, Pfeilschifter J, Bluhm J, Zschummel M, Joedicke JJ, Wirges A, et al. CXCR5 CAR-T cells simultaneously target B cell non-Hodgkin's lymphoma and tumor-supportive follicular T helper cells. Nat Commun 2021 121. 2021 Jan 11;12(1):1–19.
